# Spigelian hernia: current approaches to surgical treatment—a review

**DOI:** 10.1007/s10029-021-02511-8

**Published:** 2021-10-19

**Authors:** I. Hanzalova, M. Schäfer, N. Demartines, D. Clerc

**Affiliations:** Department of Visceral Surgery, Lausanne University Hospital CHUV, Bugnon 46, 1011 Lausanne, Switzerland

**Keywords:** Hernia surgery, Spigelian hernia, Review, Minimally invasive surgery, Surgical anatomy

## Abstract

**Background:**

Spigelian hernias (SpH) belong to the group of eponymous abdominal wall hernias. Major reasons for diagnostic difficulties are its low incidence reaching maximum 2% of abdominal wall hernias, a specific anatomical localization with intact external oblique aponeurosis covering the hernia sac and non-constant clinical presentation.

**Methods:**

A literature review was completed to summarize current knowledge on surgical treatment options and results.

**Results:**

SpH presents a high incarceration risk and therefore should be operated upon even if the patient is asymptomatic. Both laparoscopic and open repair approaches are validated by current guidelines with lesser postoperative complications and shorter hospital stay in favour of minimally invasive surgery, regardless of the technique used. Overall recurrence rate is very low.

**Conclusion:**

All diagnosed SpH should be planned for elective operation to prevent strangulated hernia and, therefore emergency surgery. Both open and laparoscopic SpH treatment can be safely performed, depending on surgeon’s experience. In most cases, a mesh repair is generally advised.

## Introduction

A Spigelian hernia (SpH) is a rare type of ventral hernia, principally acquired. Its name originates from a Flemish anatomist and physician, Adriann van den Spieghel (1578–1625), who introduced several new anatomic descriptions. Particularly, he described the well-known linea semilunaris, originally named as the *linea semilunaris spigelii* [1]*.* However, the first description of SpH itself occurred about one century later by the Bohemian anatomist and surgeon Josef Klinkosh, describing a ventral hernia occurring at the level of the “*linea spigelii*”, therefore named Spigelian Hernia. [2]. The particular anatomy of SpH contributes to the choice of the most appropriate surgical therapy, especially when it comes to minimally invasive techniques. The main objective of this review is to summarize the current evidence on the best surgical approaches for SpH, in both elective and emergency situations.

## Methods

A literature review on SpH was performed by two authors (IH, DC) using Medline, Web of Science Google scholar for English and French sources. All possible variants of Spiegel's name spelling (*“Spiegel”, “Spigelian”, “Spiegelian”, “Spigel”*) as well as both descriptive names—“*Spontaneous lateral ventral hernia”* and “*Hernia of the semilunar line”* were used. All retained studies had a special focus on surgical anatomy, surgical treatment and outcomes. Guidelines of relevant associations, such as the European hernia society (EHS) and Americas Hernia Societies (AHS) were also considered. Emphasis was put on articles published within the past 10 years.

### Surgical anatomy

**Fig. 1:**
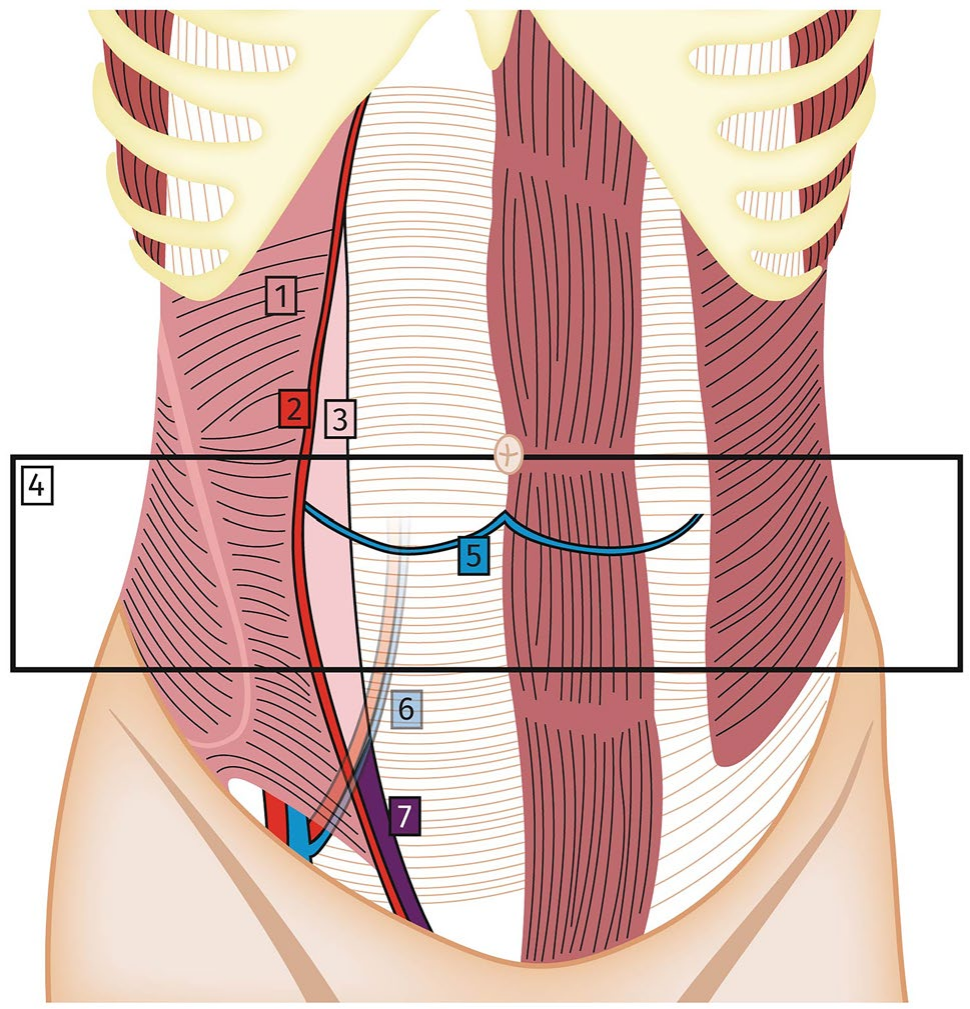
Surgical anatomy of the abdominal wall. Abdominal wall anatomy, coronal view. (1)Transverse abdominal muscle, (2) Semilunar line, (3) Spigelian fascia, (4) Spigelian hernia belt, (5) Arcuate line, (6) inferior epigastric artery and vein, (7) Low Spigelian hernia area

The SpH orifice is localised at the Spigelian point, Spigelian fascia or interchangeably called Spigelian aponeurosis, which lies laterally to the rectus abdominis muscle and medially to the semilunar line. The term semilunar line refers to the edge of the transverse and internal oblique muscles aponeuroses, respectively [3]. Hence, the semilunar line represents the border between the aponeurosis and the transverse abdominal muscle extending from the cartilage of the ninth rib to the pubic tubercle. Given a transition between Spigelian fascia and the posterior sheath of the rectus abdominis which varies above and below the arcuate line, the most common localisation of the SpH lies within an imaginary 6 cm wide band superior to the interspinous line, the so-called Spigelian belt [4] (Fig. 1). This region carries the greatest abdominal circumference and thus highest intraabdominal pressure. Consequently, the Spigelian fascia extends to its largest size that is prone to hernia development. In a recent series, Spigelian hernias located cranially to the arcuate line were reported in more than 10% of patients [5]. Furthermore, the term “*Low Spigelian hernia*” qualifies a SpH subtype located caudally to the inferior epigastric artery, within the Hesselbach’s triangle, first introduced by Spangen [4], and found in about 8% of groin hernia repairs in the Klimopoulos series [6].

**Fig. 2:**
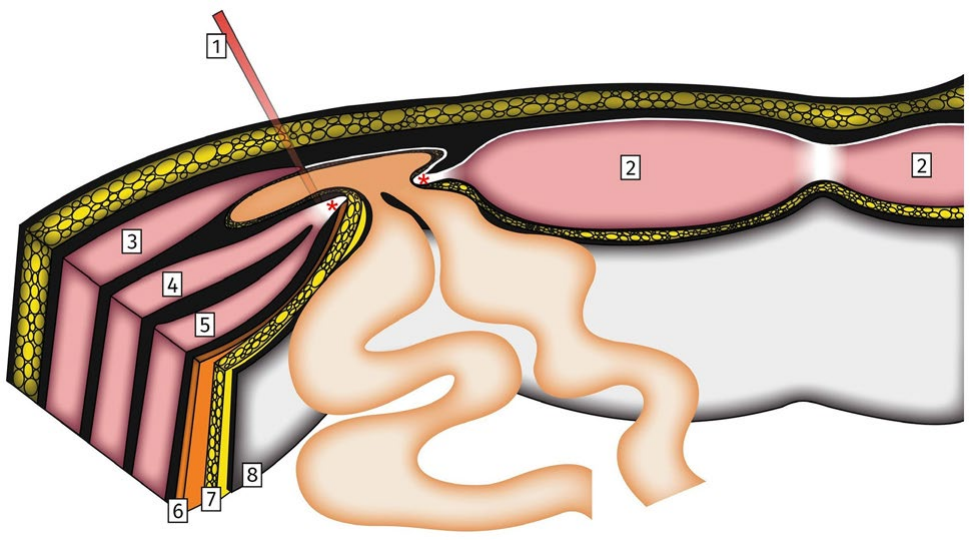
Spigelian Hernia surgical anatomy. Drawing depicting a left-sided Spigelian hernia, axial view. Note the Spigelian hernia penetrating the Spigelian fascia (red asterisks) with an intact External oblique aponeurosis. (1) Semilunar line, (2) Rectus abdominis muscle, (3) External oblique muscle with aponeurosis, (4) Internal oblique muscle, (5) Transverse abdominal muscle, (6) Fascia transversalis, (7) Pre-peritoneal fat, (8) Peritoneum

On the axial plane, the hernia sac tends to spread laterally to the interstitial zone between internal and external oblique muscles. In the majority of cases, SpH develops without penetrating the intact External oblique aponeurosis. This observation was confirmed in 90% of SpH in a French series of 51 patients [7]. The SpH specific anatomic location, with a rather small hernia orifice, usually non-exceeding 2 cm in diameter, within a rigid Spigelian aponeurosis and an inter-oblique hernia sac development make this condition difficult to diagnose with a particular tendency for incarceration (Fig. 2).

### Epidemiology

The true incidence of SpH in the general population remains unknown as many patients will never have symptoms that lead to the diagnostics. Previous reports suggests an incidence of 1–2% of all ventral hernias [5, 8]. In one large ultrasonography study of 785 anterior abdominal wall hernias, 1.4% SpH were diagnosed [9]. A recent study, searching for occult ventral hernia during general laparoscopy procedures in more than 200 patients, a Spigelian fascia defect was identified in 2% of asymptomatic patients [8]. According to the largest published series, SpH mainly affects the adult population, with a median age of 65 years at diagnosis [5, 10]. SpH is more frequent in female, with a reported female to male ratio of 2:1 [5, 11, 12]. Previous pregnancies and higher intraabdominal pressure during delivery have been assumed as predisposing factors [13, 14]. A “natural progression” of SpH in adults was recently supported by the finding that younger patients presented smaller Spigelian fascia defects with only preperitoneal content in most cases, in contrast to elderly patients who were more prone to present bigger hernias with peritoneal content at diagnosis [5]. SpH seems slightly predominant on the left side, based on a systematic review comprising more than 200 SpH [15], but underlying reasons are yet unknown.

The most important epidemiologic feature lies in the risk of incarceration with need for emergency surgery, reaching up to 24% of all SpH [4], and confirmed with contemporary data of 17% [5, 10]. This is mostly explained by a small and tight defect in the Spigelian fascia in comparison to the hernia content [5, 10, 16]. SpH carries therefore a much higher risk of incarceration compared to other ventral hernias. For instance, a rate of only 4% 5-year strangulation risk during “watch and wait” observation period of umbilical hernias is reported [17]. For inguinal hernia, the strangulation risk varies between 0.27 and 2.5% depending on the length of follow-up [18].

### Clinical presentation and differential diagnosis

The clinical presentation is frequently ambiguous and its diagnosis remains difficult. Patients most frequently experience intermittent pain, and swelling sensation in the lower abdomen [15]. According to the experience of Larson et al*.* [10], two-thirds of the patients describe clinical symptoms without any clinical findings. Webber et al*.* suggested the development of SpH in two stages: firstly, small SpH without any peritoneal component, typically occurs in younger patients complaining only of intermittent pain, without palpable mass; and secondly, a larger SpH develops with a palpable hernia sac [5]. Patients presenting evocative symptoms without any clinically palpable lump can be diagnosed having an occult SpH. Most frequently, incarcerated structures are small bowel, greater omentum and sigmoid colon [5]. Incarceration of unusual hernia content was reported in several case reports, such as Meckel's diverticulum, stomach, ovary or urinary bladder [39–41].

Low SpH can be mistaken with inguinal hernia, and final diagnosis is only confirmed intraoperatively [5, 6]. A digital palpation of the patient’s inguinal canal with Valsalva manoeuvre in a standing position has been proposed to distinguish between low SpH and direct inguinal hernia [6]. Co-existence of low SpH and direct inguinal hernia has been reported, most likely due to concomitant weakness of the Spigelian fascia around insertion of the rectus abdominis [19].

Differential diagnosis of a palpable mass in the typical region of SpH, includes lipoma [5], hematoma of rectus abdominis muscle or any abdominal solid tumour. Pain in the left inguinal fossa without palpable lump, can be confused with all other causes of left-sided abdominal pain, such as acute sigmoid diverticulitis for instance [20].

### C*lassification*

SpH is a subgroup of primary ventral hernias, and the EHS classification should be applied for classification purposes [21]. The most commonly reported hernia defect size is up to 2 cm diameter, which corresponds to the small hernia group. Medium and large SpH are those with a defect of > 2 cm and > 4 cm, respectively. A distinction between SpH with and without peritoneal components was recently described, which has an impact on the choice of the appropriate surgical approach [5]. Low SpH can be classified in the Nyhus (type Ib) or Gilbert (type 5) inguinal hernia classifications [6].

### Predisposing factors

Similar to other ventral hernias, factors contributing to high intraabdominal pressure, such as chronic obstructive pulmonary disease, chronic cough and obesity are found regularly among patients with SpH. A possible contribution of the pneumoperitoneum during a laparoscopic procedure in development of the SpH through a pre-existing weakness in the Spigelian fascia has been hypothesized by Slakey et al*.*, describing one case of the incarcerated Spigelian hernia following laparoscopic living donor nephrectomy [22]. SpH are often diagnosed in patients presenting another type of the ventral hernia, either current or previous [15, 23]. Continuous ambulatory peritoneal dialysis has also been reported as a risk factor [11, 24].

### Diagnostic workup

**Fig. 3:**
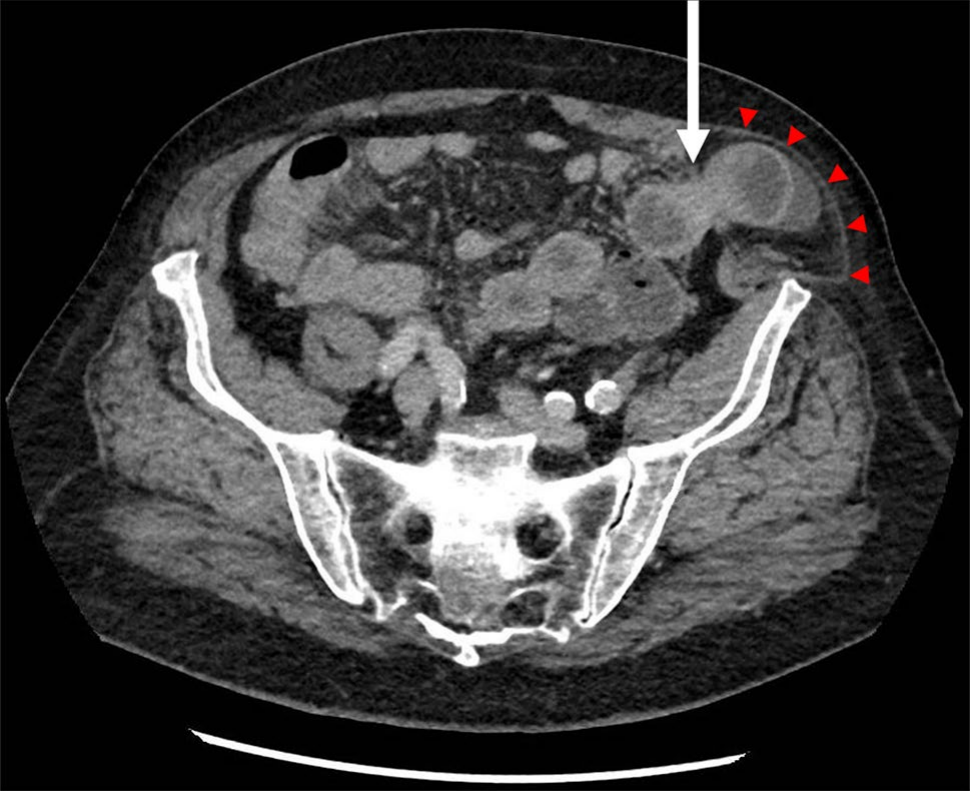
Spigelian Hernia on CT scan. Abdominal CT-scan depicting left-sided incarcerated Spigelian Hernia with small bowel content (arrow). Note the intact External oblique aponeurosis (arrowheads)

SpH remains a clinical diagnosis, but equivocal clinical features are common. Imaging techniques, such as abdominal wall ultrasonography (US) or computed tomography (CT) scan are usually performed in case of doubtful diagnosis. If doubts persist despite imaging, a diagnostic laparoscopy is considered. In the largest series published to date, up to 20% of diagnosis was confirmed by imaging method, due to unclear physical examination [10]. Several studies suggest that abdominal wall ultrasonography is a conclusive imaging method in SpH, with a sensitivity of 83–90% [24–26]. The benefit of CT scan imaging lies in the possibility to visualise the hernia content supplementary to the visualization of the hernia defect (Fig. 3). In case of an occult SpH, a diagnosis confirmation with imaging is recommended [25]. Diagnostic laparoscopy is reserved for patients with persisting symptoms and ambiguous findings on US and CT imaging [27, 28].

### Treatment options

The significant risk of incarceration taken into account, all SpH should be repaired surgically [5, 10, 29]. According to the recent EHS guidelines [28], there are no definitive preferences between open and minimally invasive approaches and the choice is at the discretion of the operating surgeon. Furthermore, a mesh repair is recommended regardless of the approach used. Repair with direct suture can be safely performed for SpH with small defects. Overall recurrence rate is very low, especially after mesh repair [23, 30]. In cases of intraperitoneal mesh placement, the use of composite or covered mesh is advocated, to decrease post-operative adhesions [31]. As for all ventral hernia, a mesh overlap exceeding the hernia neck more than 5 cm is mandatory. Closure of the SpH defect before mesh insertion is debated. While some authors consider it unnecessary [31], this issue is not addressed in the EHS position statement [28]. For most mesh repairs and regardless of the technique used, a polypropylene mesh can be inserted, except in cases of intra-abdominal mesh placement, where composite mesh should be considered [23, 34].

The most frequent early complications described are seromas, hematomas, and wound infections. While overall postoperative complications are rather rare [15, 29], they were even less frequent in case of laparoscopic repair, with 2.3% of post-operative complications reported by Skouras et al. [30]*,* compared to 18% in open cases [11]. Both studies reported a rather small number of patients and only six patients represented the relatively high rate of complications.

#### Open vs. laparoscopy

First laparoscopic operation for SpH was performed in 1992 [32]. Nowadays, minimally invasive techniques are considered and applied in all aspects of hernia surgery, aiming to reduce early postoperative morbidity, length of hospitalisation and returning earlier to normal activities. This was confirmed in cases of SpH in several series [11, 23]. Intraperitoneal laparoscopic approach gives the advantage to easily examine the entire abdominal wall while a standard open repair might require a longer incision to explore the abdominal wall, especially when the SpH is not palpable [31].

Despite evolution in minimally invasive techniques, there are still some persistent benefits to the open repair. Related to SpH size, a small preperitoneal SpH with no peritoneal sac and content, can be difficult to detect by laparoscopy. On the other hand, voluminous SpH with awaited substantial abdominal wall repair is probably best treated with an open approach [5]. Furthermore, treatment under loco-regional anaesthesia is possible only for open repair [11]. Anterior hernioplasty with pre-peritoneal sublay mesh under local anaesthesia was reported as a successful outpatient procedure without any long-term recurrences [33].

Results of some of the largest series on SpH repair published to date are summarized in Table 1.

**Table 1:** Relevant published series on Spigelian hernia surgical treatment

Year	Design	Author	Patients	Open	MIS	LOS, days	Morbidity, *n* (%)	Recurrence, *n* (%)	Follow-up, months
2002	RCT	Moreno-Egea et al	22	11	11 (8*; 3**)	Open: 3 MIS: 1*; 1,4^**^	Open: 4 (18%) MIS: 0	0	40
2002	Retrospective	Larson et al	76	75	1**	N/A	8 (11%)	Open:3 (4%) MIS: 0	96
2006	Prospective	Palanivelu et al	8	0	8***	1,2	0	0	41
2006	Prospective	Malazgirt et al	34	31	3***	4,1	6 (18%)	N/A	30
2010	Retrospective	Nirmal et al	6	0	6***	1.2	1 (17%)	0	6
2012	Retrospective	Perrakis et al	16	15	1**	Open: 3.5 MIS: 1 ^**^	2 (13%)	0	98
2012	Prospective	Zuvela et al	8	8	0	0^Δ^	0	0	23,5
2014	RCT	Moreno-Egea et al	16	0	16 (7*; 9**)	1	0	0	48
2017	Retrospective	Webber et al	101	68	33	N/A	N/A	N/A	N/A
2018	Retrospective	Rankin et al	33	27	6***	Elective: 1.6 Emergency: 5.6	7 (21%)	0	32
2020	Retrospective	Ruiz de la Hermosa et al	39	30	9**	Elective: 2.6 Emergency: 4	2 (5%)	2 (5%)	N/A

*RCT* Randomized controlled trial, *LOS* Length of hospital stay, *MIS* Minimally Invasive Surgery.

*TEP—Totally ExtraPeritoneal

**IPOM—IntraPeritoneal Onlay Mesh

***TAPP—TransAbdominal PrePeritoneal, ^Δ^Outpatient procedures

#### The choice of the laparoscopic technique

Similarly to the treatment of other ventral or inguinal hernias, minimally invasive techniques of IPOM (IntraPeritoneal Onlay Mesh), TEP (Totally ExtraPeritoneal) and TAPP (TransAbdominal PrePeritoneal) can be used for the SpH repair. There is currently no solid recommendation in favour of one of those methods but some benefits and possible limitations have been described. The only existing prospective study [31] comparing TEP versus IPOM repair demonstrated excellent results with no complications and recurrences at 4 years, for both methods. The TEP approach nevertheless reported to double the overall cost compared to IPOM, mostly due to the price of the balloon dissector.

The TEP repair is preferred by some authors as it does not require access to the peritoneal cavity and decreases the risk of intra-abdominal adhesions [27]. Moreno-Egea et al. proposed a TEP repair in patients with reducible low SpH of rather small size [31]. TEP techniques requires more technical skills with a longer learning curve compared to other laparoscopic techniques [31]. Moreover, the TEP approach can be used only if the SpH is located below the arcuate line [5]. The possible benefit from a TEP repair is the ability to explore and treat a concomitant direct inguinal hernia [19].

Advantages of an IPOM procedure, and also to the TAPP method, is the possibility to explore the whole abdominal cavity [23, 30, 34]. Both methods have also been described in the emergency setting with incarcerated hernia content. The IPOM technique is considered to be the easiest to learn and to perform safely even for surgeons not specialized in abdominal wall hernia surgery [31].

The main limitation of the IPOM is the risk of the nerve entrapment or hematoma as a result of tack or staple application. As a possible solution, a fibrin sealant instead of tacks has been proposed [35]. However, no published data on IPOM for SpH described tacks or intraperitoneal mesh complication [31]. According to Moreno-Egea et al., a SpH size of > 3 cm, bilateral hernia and irreducible content are factors in favour of an intraperitoneal technique.

One possible limitation linked to the TAPP method, highlighted by Moreno-Egea et al. [31], is the potential difficulty of the closure of the peritoneal flap, due to a thinner and fragile peritoneum in this location.

Single-incision laparoscopic totally extra peritoneal (SIL-TEP) repair of SpH was reported as an alternative technical approach. The main advantage highlighted was the cost reduction thanks to the telescopic extra peritoneal dissection, instead of standard balloon dissector used for the TEP technique. The authors described no recurrence after a short follow-up of 9 months [19].

Case reports on robotic SpH repair have been published recently, with promising preliminary data [36, 37].

#### Emergency surgery

There is a relatively small body of literature specific to the emergency surgery of SpH. Emergency SpH cases were described in case reports. In the Larson et al. series, describing eight patients for emergency clinical presentation, seven had incarcerated tissue needing a resection [10]. In this particular context, the decision to perform a mesh repair versus direct suture must be taken after considering the risk of wound infection.

## Conclusion

SpH incidence is currently unknown, but number of cases reported has recently grown with the widely use of cross-sectional imaging techniques. SpH remains a diagnostic challenge, due to the specific anatomic localisation under the external oblique aponeurosis. Moreover, SpH are significantly at higher risk of incarceration compared to other type of abdominal wall hernias. Consequently, even asymptomatic SpH should be considered for elective repair. Nowadays, and similarly to other ventral hernia, SpH can be treated with minimally invasive techniques, even in the emergency setting. There are however, no clear guidelines in favour of one specific laparoscopic technique over another with generally low morbidity and recurrence rate, based on several series of patients. An open approach remains a valid option for large hernias or in case of emergency surgery with expected bowel resection. At present, none of the existing mini-invasive approaches showed superiority, apart some anatomic limitations with the TEP approach. Intraabdominal laparoscopic approaches are easier to learn and perform with better overview of the abdominal cavity. Regardless of the technique employed, a mesh repair is recommended. Nevertheless, a non-mesh repair remains a reasonable alternative in small hernias.

The evidence regarding optimal management of SpH is still based on indirect evidence from studies with a rather limited number of patients. As a rather rare ventral hernia, further evidence on all aspects of SpH might arise from large international registry-based data.

## Data Availability

Not applicable.

## Abbreviations

SpH: Spigelian hernia
US: Ultra-sonography
CT: Computed tomography
EHS: European Hernia Society
AHS: Americas Hernia Societies
IPOM: IntraPeritoneal onlay mesh
TEP: Totally extraperitoneal
TAPP: TransAbdominal PrePeritoneal
SIL: Single incision laparoscopy
